# A simple protocol to establish a conditionally immortalized mouse podocyte cell line

**DOI:** 10.1038/s41598-024-62547-5

**Published:** 2024-05-21

**Authors:** Yujiao Huang, Jie Geng, Mengdan Wang, Wenbin Liu, Haikun Hu, Wei Shi, Mei Li, Guiyang Huo, Guangrui Huang, Anlong Xu

**Affiliations:** 1 School of Life Sciences, Beijing University of Chinese Medicine, Beijing, 102488 China; 2 Dongfang Hospital of Beijing University of Chinese Medicine, Beijing, 100078 China

**Keywords:** MPC, Podocyte injury, Suckling mouse, Synaptopodin, WT1, Biotechnology, Cell biology, Nephrology

## Abstract

Podocytes are specialized terminally differentiated cells in the glomerulus that are the primary target cells in many glomerular diseases. However, the current podocyte cell lines suffer from prolonged in vitro differentiation and limited survival time, which impede research progress. Therefore, it is necessary to establish a cell line that exhibits superior performance and characteristics. We propose a simple protocol to obtain an immortalized mouse podocyte cell (MPC) line from suckling mouse kidneys. Primary podocytes were cultured in vitro and infected with the SV40 tsA58 gene to obtain immortalized MPCs. The podocytes were characterized using Western blotting and quantitative real-time PCR. Podocyte injury was examined using the Cell Counting Kit-8 assay and flow cytometry. First, we successfully isolated an MPC line and identified 39 °C as the optimal differentiation temperature. Compared to undifferentiated MPCs, the expression of WT1 and synaptopodin was upregulated in differentiated MPCs. Second, the MPCs ceased proliferating at a nonpermissive temperature after day 4, and podocyte-specific proteins were expressed normally after at least 15 passages. Finally, podocyte injury models were induced to simulate podocyte injury in vitro*.* In summary, we provide a simple and popularized protocol to establish a conditionally immortalized MPC, which is a powerful tool for the study of podocytes.

## Introduction

Podocytes are specialized terminally differentiated cells that encompass the outer surface of the glomerular basement membrane via intricate interdigitating foot processes and play a pivotal role in the renal filtration process^1^. Formation of the slit diaphragm (SD), which is an interlocking structure between adjacent foot processes, is a vital component of the filtration barrier that effectively prevents proteinuria^2^. Podocytes face a multitude of physiological stresses within their complex environment, including mechanical strain and chemical stimuli^3^. Despite their powerful adaptive mechanisms, podocytes may succumb to maladaptation and injury under stress, which contribute to various glomerular diseases. Exposure to high glucose (HG) induces podocyte apoptosis, which leads to extensive proteinuria in diabetic nephropathy (DN)^4–6^. Podocytes become targets of autoimmune responses, and the interaction between antigens and antibodies results in the loss of podocyte structure or function. This immune-mediated assault on podocytes has led to an increasing incidence of podocyte-related glomerular diseases, such as minimal change disease and membranous nephropathy^7,8^. Therefore, the refinement of tools to study podocyte biology is imperative for a comprehensive understanding of their underlying mechanisms.

Previous methodologies successfully established temperature-sensitive human and mouse podocyte cell lines. The temperature-sensitive simian virus 40 large T antigen (SV40 TAg) protein confers cell immortality and facilitates cell proliferation at lower temperatures (33 °C) while inducing differentiation at higher temperatures (37–39.5 °C)^9–11^. Researchers created human podocyte cell lines by infecting primary podocytes from healthy individuals with a retrovirus containing the SV40 tsA58 gene^12^ and obtained a mouse podocyte cell line from *H-2Kb-tsA58* transgenic mice^13,14^. These established podocyte lines have made outstanding contributions to global podocyte research and significantly enhanced our understanding of kidney disease mechanisms^15–20^. Due to the challenges associated with obtaining human renal samples, the absence of suitable animal models for certain kidney diseases, and ethical considerations, some mechanistic studies cannot be performed in vivo. Therefore, it is necessary to use in vitro cultured primary podocytes and immortalized podocyte cell lines for research purposes. The differentiation period for existing immortalized podocytes typically ranges from 7 to 14 days^21,22^. While the in vitro lifespan of differentiated podocytes is generally 2–3 weeks, this duration may be extended to 28 days by modifying the culture surface^23^. The protracted differentiation period and limited lifespan of podocytes in vitro negatively impact experimental efficiency and impede progress. The stability of podocyte-specific protein expression diminishes as the number of cell passages increases. When establishing immortalized podocyte cell lines, the initial step involves extracting primary podocytes from the glomeruli. Various methods for glomerular extraction have been reported, including the differential adhesion method^24^, the perfusion sorting method using Dynabeads (Ф4.5 µm)^25^, and the modified method using Dynabeads (Ф4.5 µm) mixed with iron powder (Ф6 µm)^26^. However, these methods include intricate steps and are not easily applicable in general laboratory settings. Therefore, there is an urgent need to establish a simple and widely applicable method for establishing podocytes in laboratory settings. It is crucial to establish podocyte cell lines with shorter differentiation periods and extended lifespans in vitro.

The kidneys of suckling mice continue to grow during the first week after birth, but their growth rate slows after the third week^27^. Postnatal growth of the nephron density in mice is faster than during the prenatal period^28^. Therefore, suckling mice have a significantly greater glomerular density compared to mature mice. Due to incomplete development, suckling mice also have a limited number of renal tubules. The glomeruli of suckling mice also tend to adhere to culture dishes, which leads to a longer in vitro survival period of podocytes^29^. Therefore, by harnessing the unique characteristics of suckling mouse glomeruli and cells, it is possible to establish an improved conditionally immortalized podocyte cell line.

We propose a simple protocol for obtaining immortalized mouse podocyte cell (MPC). To enhance the feasibility of culturing primary podocytes and improve their adaptability to in vitro conditions, we selected suckling mice as the source of glomeruli. The glomeruli from suckling mice were isolated through a cell strainer, followed by lentivirus infection after in vitro culture. Monoclonal cell lines were subsequently selected and expanded, and MPCs were identified using RT-qPCR analysis. Notably, we determined that a temperature of 39 °C was more conducive to MPC differentiation compared to 37 °C. Following differentiation, the expression of WT1 and synaptopodin was upregulated in MPCs, and normal expression of podocyte-specific proteins was maintained for at least 15 passages. We successfully induced in vitro models of podocyte injury using HG, adriamycin (ADR), and lipopolysaccharide (LPS). In conclusion, we present a streamlined and widely applicable protocol for establishing conditionally immortalized MPCs as a potent tool for investigating glomerular diseases.

## Materials and methods

### Mice

The C57BL/6 J mice used in this study, aged 7–8 weeks and 7 days, were obtained from Beijing Vital River Laboratory Animal Technology Co., Ltd. (Beijing, China). Mice were euthanized with isoflurane. All animal experiments were reviewed and approved by the Beijing University of Chinese Medicine Animal Care and Use Committee (ethics number BUCM-4-2022071501-3014). The study was reported in accordance with ARRIVE guidelines. All of the following methods were performed in accordance with the relevant guidelines and regulations.

### Establishment of the conditionally immortalized MPCs

#### Isolation of glomeruli from the kidneys of suckling mice

The kidneys were harvested from suckling mice (7 days old) and finely minced into 1 mm^3^ fragments within a 6-cm cell culture dish (Corning, USA). The tissue fragments were enzymatically digested using a collagenase solution comprised of 1 mg/mL collagenase IV (Worthington Biochemical, USA) and 0.002 U/mL DNase I (Biorigin, China) in Hanks’ balanced salt solution (HBSS) at 37 °C for 5 min with gentle agitation. The digestion process was terminated by the addition of fetal bovine serum (FBS) (Sigma, USA). The collagenase-digested tissues were gently pressed through a 70-µm cell strainer (BD Falcon, USA) using the rubber plunger of a 5-mL syringe. The strainer was rinsed with HBSS to ensure maximal recovery. The entire filtrate was collected and subjected to centrifugation at 1000×*g* and 4 °C for 5 min. The isolated glomeruli were cultured in 6-cm cell culture dishes at 37 °C using 5 mL of RPMI 1640 medium (Corning, USA) supplemented with 10% FBS (Sigma, USA) and 1% penicillin‒streptomycin (Vetec, USA). After a two-day incubation period, the glomeruli adhered to the dish, and the medium was replenished. Microscopy revealed the outward spread of the outer layer of glomerular cells.

#### Isolation and culture of primary podocytes in vitro

Once the glomeruli attached and reached 80% confluence, the cells were carefully dissociated using 0.25% trypsin–EDTA (Gibco, USA). The resulting digestion solution was filtered through a 40-µm cell strainer (BD Falcon, USA). Primary podocytes were cultured in a 6-cm cell culture dish, and the medium was changed every other day. Microscopy assessed the morphology and degree of cellular confluence. The cells were used for the in vitro identification of podocytes once the confluency reached 80%.

#### Lentivirus infection

The podocyte suspension was diluted to a concentration of 2 × 10^5^ cells/mL, and 500 µL/well of the diluted suspension was inoculated into a 24-well plate (Corning, USA). On the following day, podocytes were infected with a lentivirus (obtained from the Public Protein/Plasmid Library, China) carrying SV40 tsA58 and the puromycin resistance gene. The infection system was supplemented with 8 µg/mL polybrene (Beyotime, China) to enhance the efficiency of viral infection. The infected cells were maintained in culture at 33 °C. After an incubation period of 8–16 h, the culture medium containing the same viral titer was replaced. After 48 h, the cells were selected with 0.5 µg/mL puromycin (Solarbio, China) for an additional 48 h. Cell culture was continued until the confluency reached 90%.

### Limiting dilution and selecting the monoclonal MPC

The infected cells were cultured in a 96-well plate (Corning, USA) to isolate purified cell clones. A total of 16 clones were observed within the 96-well plate, and 13 subclones were successfully expanded. Upon reaching confluency, the subclones were sequentially passaged into 48-well plates, 24-well plates, and 6-well plates (all from Corning, USA) for expansion. These expanded cell populations were used for subsequent cell identification.

### MPC culture

Cells exhibit a cobblestone-like morphology when cultured at a permissive temperature of 33 °C. To induce podocyte differentiation and promote physiological morphology, the cells were subjected to a temperature shift from permissive conditions to nonpermissive temperatures of 37 °C or 39 °C. The resulting differentiated podocytes were used for subsequent experiments.

For HG induction, after a 12-h period of serum starvation, the differentiated podocytes were cultured at 39 °C for 24 h in the following conditions: 5 mM glucose (normal group, NG; LABLEAD, China), 5 mM glucose + 25 mM mannitol (high permeability, HP group; Merck, USA), 30 mM glucose (HG group), and 30 mM glucose + 2.5 mM or 5 mM berberine (HG + BBR group; Solarbio, China). For LPS induction (Sigma, USA), differentiated podocytes were treated with various concentrations of LPS (0, 25, 35, or 45 μg/mL) at 39 °C for 24 h. For ADR induction (Solarbio, China), differentiated podocytes were treated with different concentrations of ADR (0, 0.1, 0.3, or 0.5 μg/mL) at 39 °C for 24 h.

### RNA extraction and quantitative real-time PCR (RT‒qPCR)

The mouse renal cortex (MKC) was obtained from 6- to 8-week-old mice. Total RNA was extracted using the Steadypure Universal RNA Extraction Kit (Accurate Biology, China) and quantified using P200/P200 + Micro Volume Spectrophotometers (Pultton, USA). Total RNA was subjected to reverse transcription using the Evo M-MLV RT Premix for qPCR (Accurate Biology, China). PCR amplification was performed using the SYBR Green Premix Pro Taq HS qPCR Kit (Accurate Biology, China). Details of all of the primers used in this study are shown in Table 1. The relative expression levels of the target genes were normalized to GAPDH using the ^ΔΔ^Ct method.

**Table 1:** Primers used for RT-qPCR.

Gene	Forward primer (5′–3′)	Reverse primer (5′–3′)
*Wt1*	AGTGAAATGGACAGAAGGGCAGA	TCCAGATACACGCCGCACAT
*Synpo*	GACCAGCCAGATAGAGCAAAGC	GGGGAGACCTAACCCGAGAA
*Thsd7a*	ACCTGGGTTTATGGTGTCG	GGTTTGAGGCGGTTGTT
*Nphs1*	TGGCGATTCCTGCCTCCGTT	TTCTGCTGGGAGCCCTCGTT
*Nphs2*	GAGGATGGCGGCTGAGATTC	GGTAGTTGATGCTCCCTTGTGCT
*Slc5a2*	ATGGAGCAACACGTAGAGGC	ATGACCAGCAGGAAATAGGCA
*Scl34a1*	AGTGAATGATGTCCTACAGCGAGA	ACTGGAGATGGCATAGGTGGAT
*Fxyd2*	GACTATGAAACCGTCCGCAAAG	CCCCACAGCGGAACCTTTT
*Cldn1*	GGGGACAACATCGTGACCG	AGGAGTCGAAGACTTTGCACT
*Pax8*	AGAGTCACCCCAGTCGGATTC	GCTGGCTGAAGGTGTCCGTA
*Krt8*	CAAGGTGGAACTAGAGTCCCG	CTCGTACTGGGCACGAACTTC
*Pecam1*	CTGCCAGTCCGAAAATGGAAC	CTTCATCCACCGGGGCTATC
*Flt1*	CCACCTCTCTATCCGCTGG	ACCAATGTGCTAACCGTCTTATT
*Pdgfrb*	TTCCAGGAGTGATACCAGCTT	AGGGGGCGTGATGACTAGG
*Gata3*	GAACTGCGGGGCAACCTCTA	GCCTTCGCTTGGGCTTGATA
*SV40 tsA58*	CTTGAAAGGAGTGCCTGGGG	TCCCATTCATCAGTTCCATAGGT
*Tlr1*	TGAGGGTCCTGATAATGTCCTAC	AGAGGTCCAAATGCTTGAGGC
*Il6*	CTGCAAGAGACTTCCATCCAG	AGTGGTATAGACAGGTCTGTTGG
*Gapdh*	GCACAGTCAAGGCCGAGAAT	GCCTTCTCCATGGTGGTGAA

### Western blot

Protein was extracted from the cells, and the protein concentrations were quantified using a BCA protein assay kit (Thermo, USA). Equal amounts of protein samples were loaded and subjected to electrophoresis on an 8% sodium dodecyl sulfate–polyacrylamide gel and then transferred to polyvinylidene difluoride membranes (Millipore, USA). The membranes were blocked for 1 h at room temperature using a 5% skim milk powder solution in Tris-buffered saline with Tween-20 (TBST). The membranes were incubated overnight at 4 °C with primary antibodies (Table 2). The membranes were incubated with a horseradish peroxidase-labeled secondary antibody (Table 2) for 1 h at room temperature. After thorough washing with TBST, the membranes were visualized using an enhanced chemiluminescence (ECL) kit (CWBIO, China). The intensity of the bands was analyzed using ImageJ software (nih.gov).

**Table 2:** List of antibodies used in the study.

Antibody	Host	Application (dilution)	Supplier
Synaptopodin	Rabbit	WB (1:1000), IF (1:500)	Proteintech, 21064-1-AP
Synaptopodin	Rabbit	IF (1:200)	Abcam, ab259976
Synaptopodin	Mouse	IF (1:100)	Santa Cruz, sc-515842
Wilms tumor protein	Rabbit	WB (1:1000), IF (1:100)	Abcam, ab89901
Nephrin	Rabbit	IF (1:200)	Abcam, ab216341
PECAM1	Rabbit	WB (1:2000)	Abcam, ab222783
KRT8	Rabbit	WB (1:10,000)	Abcam, ab53280
GATA3	Rabbit	WB (1:2000)	Abcam, ab199428
SV40 TAg	Rabbit	WB (1:2000)	Abcam, ab234426
GAPDH	Rabbit	WB (1:10,000)	Abcam, ab8245
Goat anti-rabbit IgG H&L	NA	WB (1:10,000)	Abcam, ab6721
Goat anti-rabbit AF555	NA	IF (1:500)	Abcam, ab150186
Goat anti-mouse AF555	NA	IF (1:500)	Abcam, ab150114

### Indirect immunofluorescence staining

The cells were fixed with 4% paraformaldehyde (Biorigin, China) for 15 min at room temperature (RT), followed by permeabilization using 0.1% Triton X-100 in PBS (Sigma, USA) for 10 min at RT. The cells were blocked with goat serum (ZSGB-BIO, China) for 1 h at RT. After overnight incubation at 4 °C with primary antibodies (refer to Table 2), the cells were stained with fluorescently labeled secondary antibodies for 30 min at RT. DNA visualization was achieved using DAPI (Biorigin, China). The slices were mounted using Antifade Mounting Medium (Biorigin, China). Immunofluorescent staining was captured at a magnification of 40 × .

### Proliferation assay

To assess the proliferative capacity of MPCs at permissive and nonpermissive temperatures, the cells were seeded into a 24-well plate at a density of 1 × 10^4^ cells/well. The MPCs were cultured at 33 °C and 39 °C. Cell counting was performed using blood cell counting plates every 24 h for both temperature conditions.

### MPC viability assay

The viability of podocytes was assessed using a Cell Counting Kit-8 (CCK-8) (Biorigin, China). Differentiated podocytes (1.0 × 10^4^ cells per well) were seeded into 96-well plates and incubated at 39 °C. After 24 h, a mixture of CCK-8 and serum-free RPMI 1640 medium was prepared at a ratio of 1:10. The podocytes were incubated with the CCK-8 mixture for 1 h at 37 °C and 5% CO_2_. The absorbance values of each well were measured at 450 nm using an Infinite M Nano spectrophotometer (TECAN, China).

### Flow cytometry

The apoptosis of podocytes cultured under various conditions was evaluated using an Annexin V Apoptosis Detection Kit (BD Biosciences, USA), and the data were analyzed with FlowJo software. Apoptotic podocytes were defined as Annexin V-positive/PI-positive cells.

### Statistical analysis

All statistical analyses were performed using GraphPad Prism 8.0.2 and IBM SPSS Statistics 20. The data are expressed as the means ± SEM. A t test was used for statistical comparisons between two groups, and one-way ANOVA was used for comparisons of multiple groups. A *P* value < 0.05 was considered statistically significant.

## Results

### Isolation of podocytes from suckling mouse kidneys and culture in vitro

**Figure 1:**
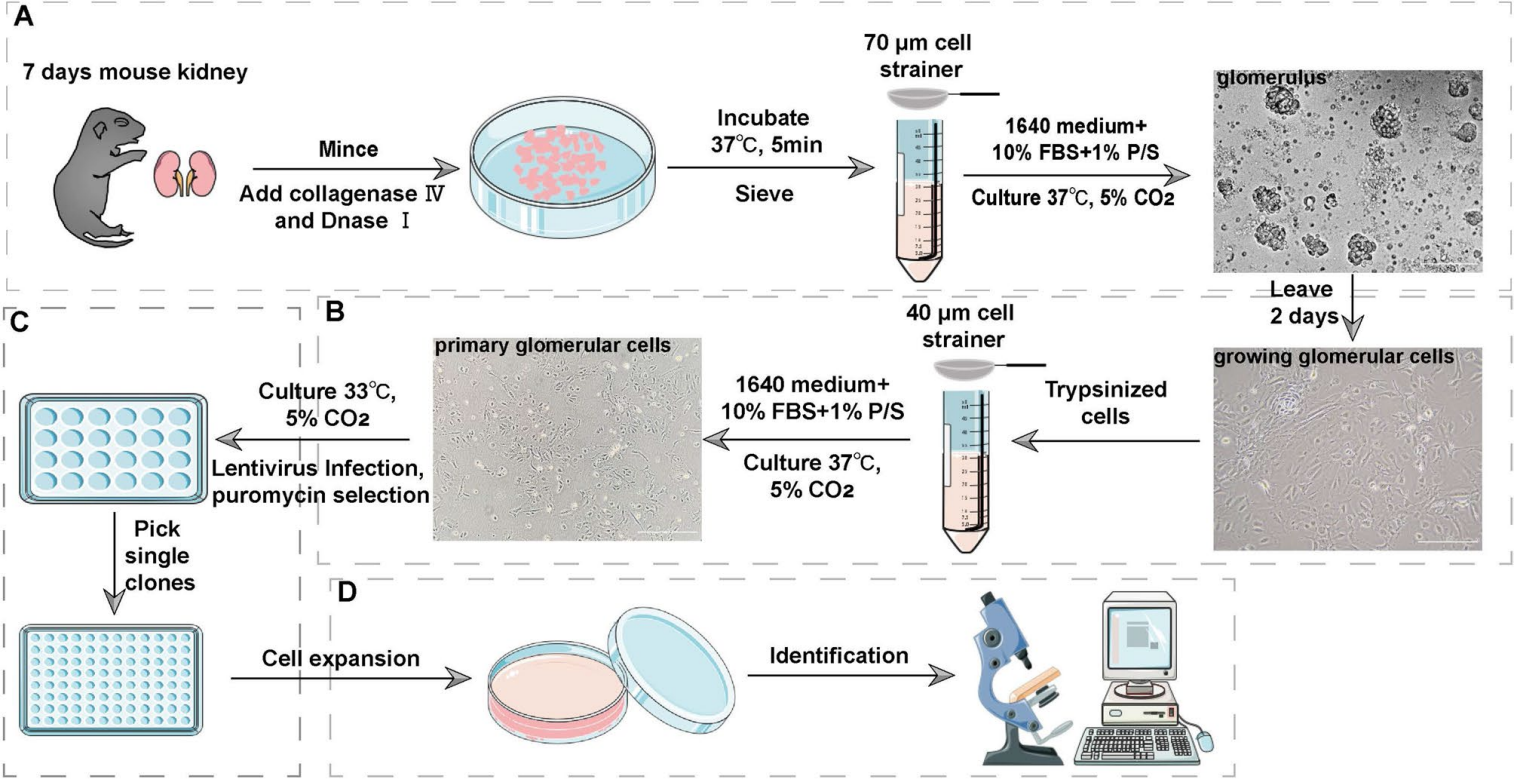
The pipeline for establishing conditionally immortalized MPCs*.* (**A**) To obtain podocytes for establishing a conditionally immortalized MPC model, glomeruli were harvested from suckling mice. Kidneys were minced to 1-mm^3^ pieces in a 6-cm cell culture dish. The tissues were digested in collagenase solution. The collagenase-digested tissues were gently pressed through a 70-µm cell strainer, and the glomeruli were cultured at 37 °C. (**B**) Two days later, outgrowing cells appeared as a monolayer around the individual glomeruli. Trypsin-digested cells were passed through a 40 µm cell strainer to remove the glomerular cores. The primary glomerular cells were cultured at 37 °C until the cell confluence reached 80%. (**C**) Podocytes were infected with lentivirus carrying SV40 tsA58 and the puromycin resistance gene. We performed limited dilutions in 96-well plates and identified and numbered individual cell wells using microscopy. (**D**) Thirteen monoclonal cell lines were successfully isolated. The 13 monoclonal cell lines were subjected to preliminary identification using RT-qPCR. A monoclonal podocyte line was used for further identification. Imaging materials were obtained from smart.servier.com. The scale is 100 µm.

To obtain podocytes for establishing a conditionally immortalized MPC, glomeruli were harvested from suckling mice. Despite the relatively smaller kidney volume of suckling mice compared to adult mice, a substantial number of intact glomeruli were successfully collected in the filtrate (Fig. 1A). After 48 h of cultivation, all glomeruli adhered to the culture dish, and the cells formed a distinct monolayer surrounding the glomerular cores (Fig. 1B). A minor fraction of tubules was also present in the cell dishes, but they did not attach under these specific culture conditions and were removed via medium changes. Once the cell confluence reached 80%, the cells were harvested and passed through a 40-µm cell strainer to eliminate the glomerular cores. The primary podocytes exhibited an arborized or cobblestone-like appearance similar to a primary culture^26,30,31^ (Fig. 1B). The cells were infected with the SV40 tsA58 gene and subjected to selection with puromycin (Fig. 1C). Ultimately, 13 monoclonal cell lines were isolated, and one monoclonal cell line was chosen for podocyte characterization (Fig. 1D).

### Podocytes were identified in primary cultured glomerular cells in vitro

**Figure 2:**
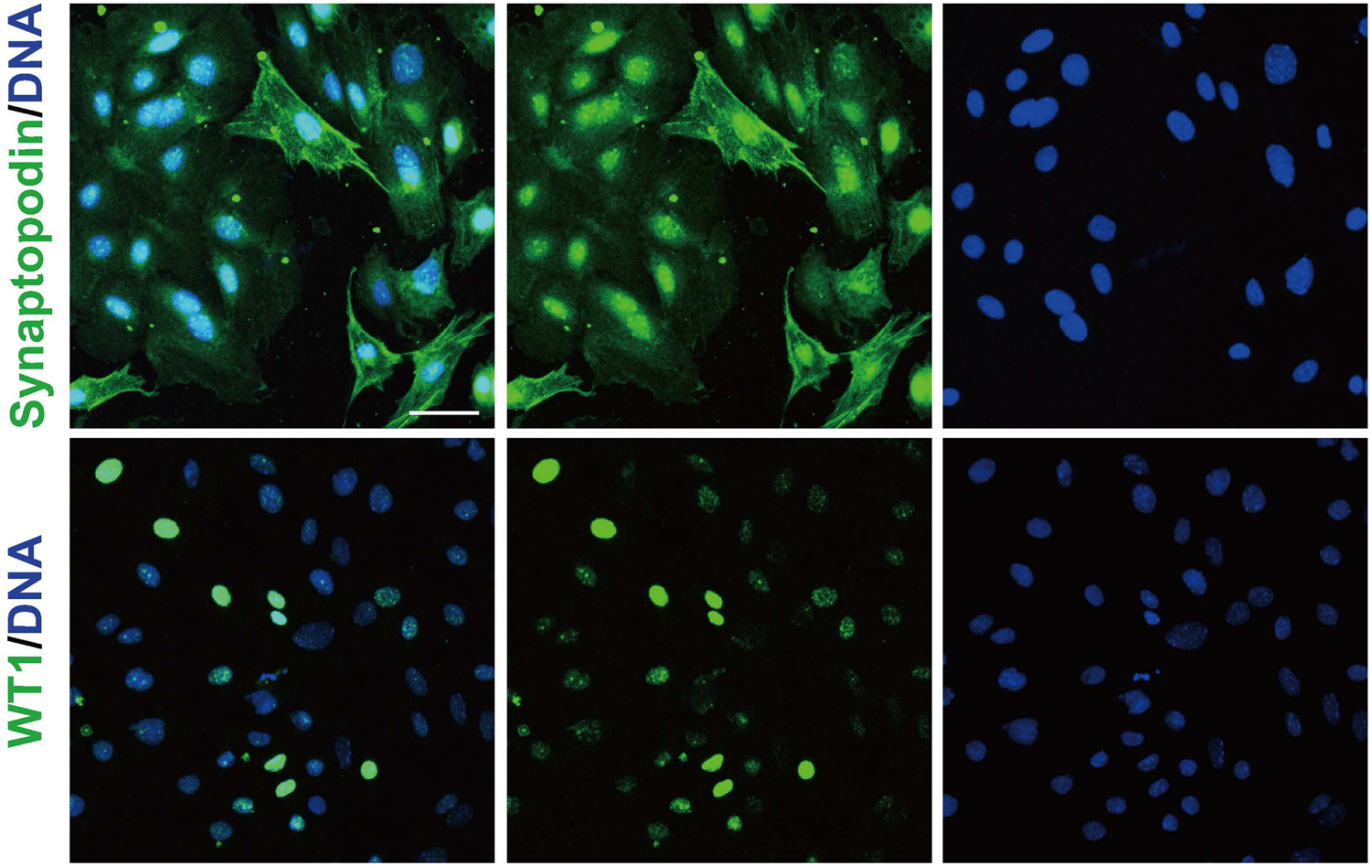
Identification of podocytes within glomerular cells cultured in vitro. Indirect immunofluorescent staining revealed strong and weak variations in synaptopodin and WT1 staining in glomerular cells. Synaptopodin showed positive staining in the cytoplasm and at the cell border. WT1 was co-localized in the nucleus with DAPI staining. The scale is 50 µm.

To verify that the cells emerging from the glomerulus contained podocytes, we detected the expression of podocyte markers in glomerular cells cultured in vitro using indirect immunofluorescence staining. Wilms tumor 1 (encoded by *Wt1*) is a pivotal transcription factor in the maintenance of mature podocytes^32,33^. Synaptopodin (encoded by *Synpo*) plays a crucial role in regulating podocyte actin dynamics and sustaining glomerular filter function by stabilizing stress fibers^34–36^. These genes are commonly used for podocyte characterization. To identify podocytes within the cultured glomerular cells, immunofluorescence staining was performed. Our results showed that Synaptopodin and WT1 were expressed in cultured cells (Fig. 2). There was less synaptopodin expression in the arborized cells than in the cobblestone-like cells, which may be due to the dedifferentiation of the cobblestone-like cells during in vitro culture^26^. In arborized podocytes, synaptopodin showed positive staining at cell borders and the cytoplasm. The expression of WT1 suggested that podocytes had different maturation states. Negative controls ensured antibody specificity. Immunofluorescence staining showed that synaptopodin was also expressed in nuclear cells. Therefore, the same experiment was performed with two other antibodies, which were also found to be expressed in the nucleus (Supplementary Fig. S7), and the results were consistent with previous studies^26,37^.

### Identification of the monoclonal MPC and its optimum nonpermissive temperature

**Figure 3:**
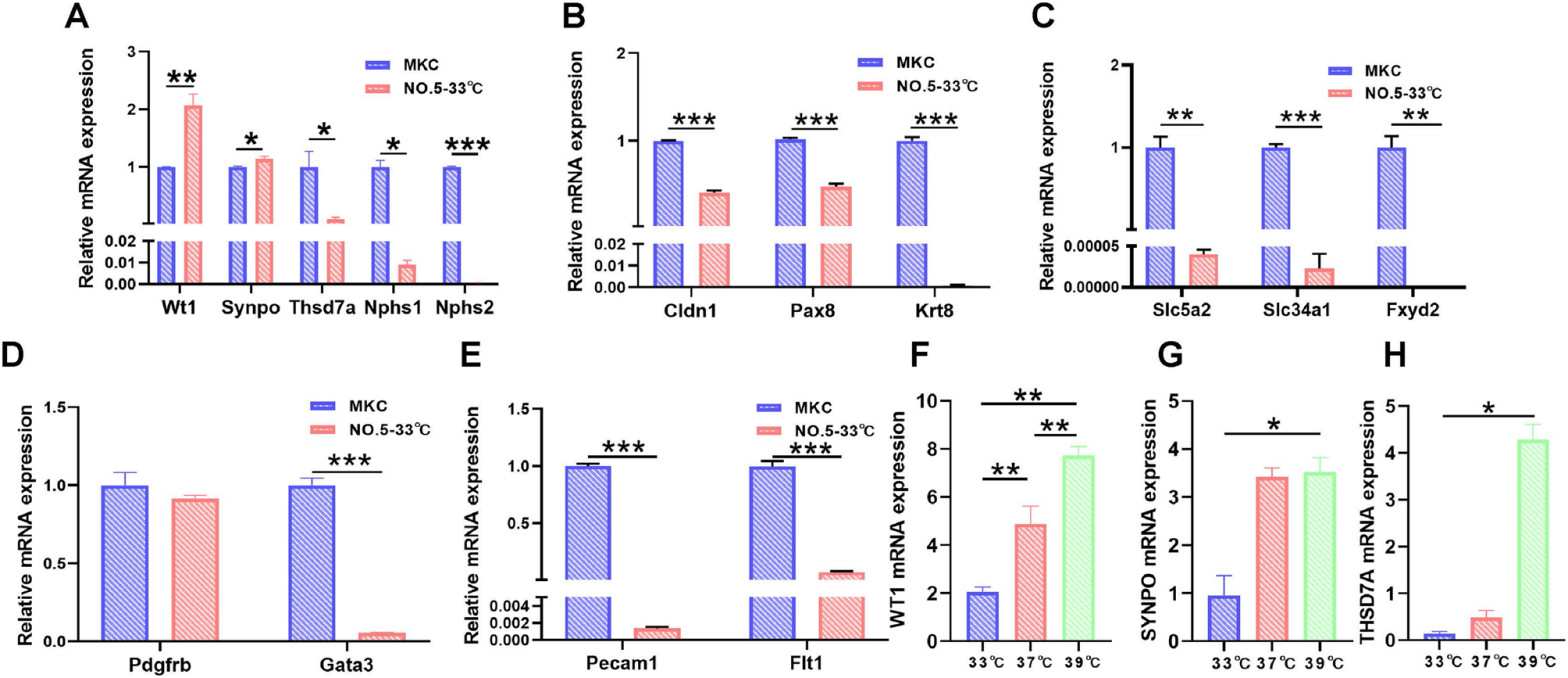
Identification of the monoclonal MPC and its optimum nonpermissive temperature. RT-qPCR was used to measure the expression of markers. (**A**) The expression of podocyte-specific genes in the No. 5 cell line, including *Wt1, Synpo, Thsd7a, Nphs1* and *Nphs2*. (**B**) The expression of PEC-specific genes in the No. 5 cell line, including *Cldn1, Pax8* and *Krt8*. (**C**) The expression of renal tubular epithelial cell-specific genes in the No. 5 cell line, including *Slc5a2, Slc34a1*and *Fxyd2*. (**D**) The expression of mesangial cell-specific genes in the No. 5 cell line, including *Padgftb* and *Gata3*. (**E**) The expression of endothelial cell-specific genes in the No. 5 cell line, including *Pecam1* and *Flt1*. Comparison with permissive temperature, RT-qPCR assays for podocyte-specific gene expression at two nonpermissive temperatures of 37 °C and 39 °C. The expression of *Wt1* (**F**), *Synpo* (**G**), and *Thsd7a* (**H**) were compared to MKCs. The data are presented as the means ± SEM (n = 3), *P < 0.05, **P < 0.01, ***P < 0.001.

For immortalization, glomerular cells were infected with the SV40 tsA58 gene. Limited dilutions were performed in 96-well plates, and the monoclonal cell lines were observed via microscopy. A total of 13 monoclonal cell lines were identified. To select podocytes from the 13 monoclonal cell lines, we detected the expression of marker genes for podocytes, mesangial cells, endothelial cells, parietal epithelial cells (PECs) and renal tubule cells. Nephrin (encoded by *Nphs1*) is a protein specifically localized to the SD^38,39^. Thrombospondin type-1 domain-containing protein 7A (encoded by *Thsd7a*) is expressed in the SD, and it promotes the migration and adhesion of podocytes and maintains the stability of podocyte membrane dynamics^40^. Podocin (encoded by *Nphs2*) regulates the stability of podocytes in the SD^39^. *Cldn1*, *Pax8*, and *Krt8* are PEC-specific genes^41–43^. *Gata3* and *Pdgfrb* are mesangial cell-specific genes^44,45^. *Pecam1* and *Flt1* are endothelial cell-specific genes^46,47^, and *Slc5a2*, *Slc34a1*, and *Fxyd2* are renal tubular epithelial cell-specific genes^48–51^. The No.5 cell line showed sufficient podocyte characteristics compared to the other monoclonal cell lines (Fig. 3A–E). The No. 1 cell line showed characteristics of mesangial cells, and the No. 13 cell line exhibited characteristics of PECs (Supplementary Figs. S1–S5). These results indicated that the No. 5 cell line was podocytes, the No. 1 cell line was mesangial cells, and the No. 13 cell line was PECs. The expression of genes specific to endothelial cells and renal tubular epithelial cells in all monoclonal cell lines was significantly lower than MKCs (Supplementary Figs. S4, S5), which indicated that there were no endothelial cells or renal tubular epithelial cells. Therefore, we identified the No. 5 cell line as a podocyte line for study.

To identify the optimum differentiation temperature, we compared the expression of *Wt1*, *Synpo*, and *Thsd7a* in MPCs at 37 °C and 39 °C. As shown in Fig. 3F–H, the expression levels of these podocyte-specific genes were higher at 37 °C and 39 °C compared to 33 °C. Compared to 37 °C, podocytes cultured at 39 °C showed higher gene expression, which indicated that 39 °C was more suitable for podocyte differentiation. Therefore, 39 °C was chosen as the optimal differentiation temperature for MPCs.

### Characteristics of MPCs

**Figure 4:**
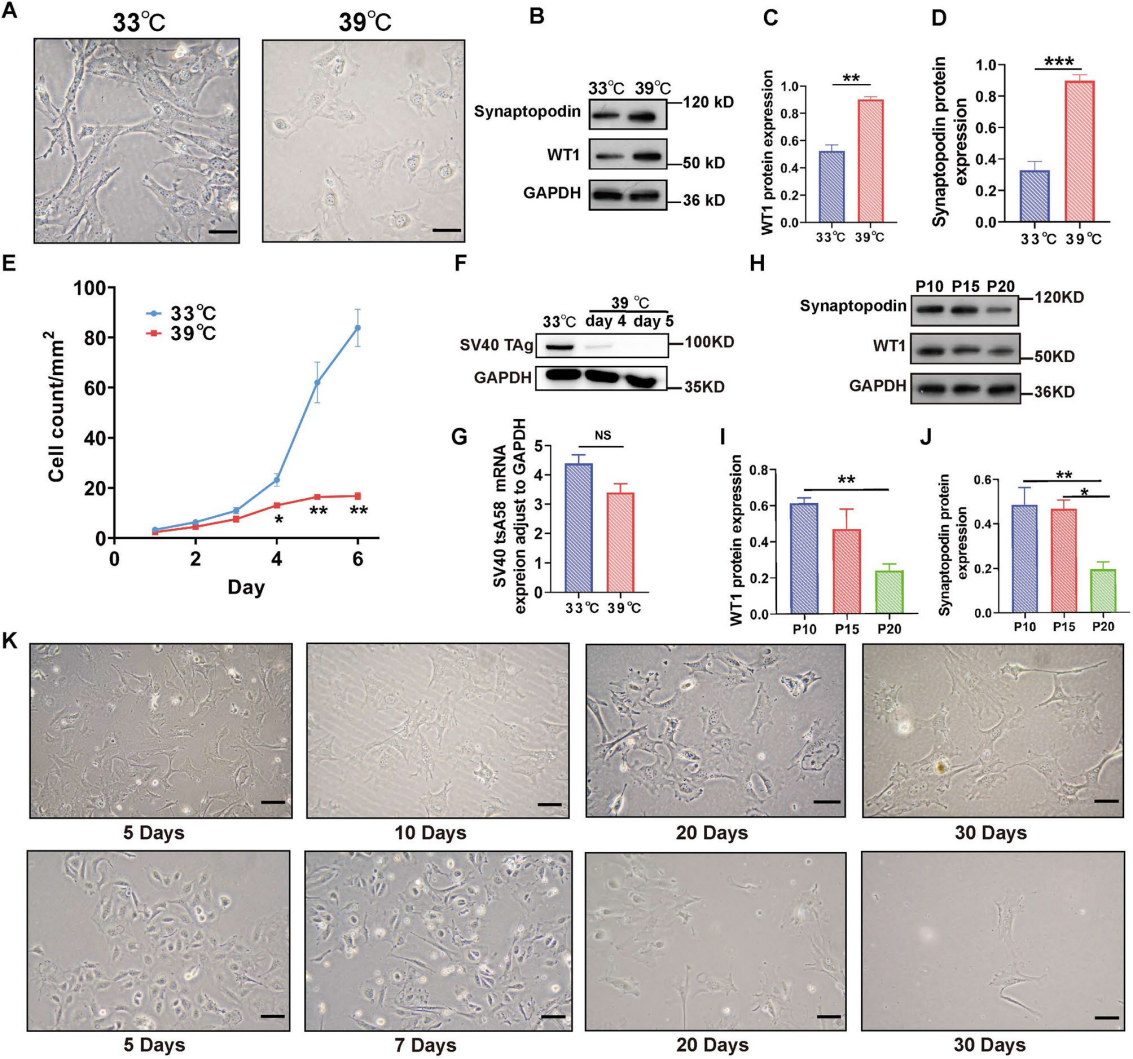
Characteristics of MPCs. (**A**) Podocyte morphology at 33 °C and 39 °C under a microscope. The scale is 50 µm. (**B**) WT1 and synaptopodin were detected at 33 °C and 39 °C using Western blot analysis. (**C**,**D**) Intensity analyses of WT1 and synaptopodin (**B**) using ImageJ. (**E**) Comparison of cell proliferation under permissive and nonpermissive temperatures. (**F**) The protein expression of SV40 TAg was detected in MPCs cultured at permissive temperature (33 °C) and nonpermissive temperature (39 °C). (**G**) The mRNA expression of the SV40 tsA58 gene did not differ between differentiated and undifferentiated MPCs according to RT‒qPCR. (**H**) Changes in WT1 and synaptopodin expression at P10, P15, and P20 in differentiated MPCs. (**I**,**J**) Intensity analyses of WT1 and synaptopodin using (**H**) ImageJ. (**K**) The growth status of the MPCs (above) and the existing mouse podocyte cell line (below) on days 5, 10, 20, and 30 was evaluated via optical microscopy at 39 °C or 37 °C. The scale is 50 µm. The data are presented as the means ± SEM (n = 3–6), *P < 0.05, **P < 0.01, ***P < 0.001.

At 33 °C, undifferentiated podocytes proliferate in a typical cobblestone morphology. At 39 °C, the differentiated podocyte volume gradually increased, and the podocytes exhibited filopodia or thin arborized protrusions (Fig. 4A). Western blot analysis revealed that WT1 and synaptopodin were significantly increased in differentiated podocytes compared to undifferentiated podocytes (Fig. 4B–D), which is consistent with the results in Fig. 3F–G. PECAM1, KRT8, and GATA3 were not expressed in MPCs (Supplementary Fig. S6). These results indicate that the morphology and protein expression of differentiated podocytes are characteristic of mature podocytes under physiological conditions.

To compare the proliferation speed of the MPCs, the cells were counted at 33 °C and 39 °C. At permissive temperatures, the number of podocytes increased continuously, and the number of undifferentiated podocytes increased exponentially beginning on the fourth day. However, at nonpermissive temperatures, the number of podocytes tended to stabilize starting on the 5th day, and there was no further increase in the cell count (Fig. 4E). SV40 TAg was strongly expressed in MPCs at 33 °C. In contrast, after 5 days at 39 °C, the expression of this protein was not detected (Fig. 4F). This observation suggested that cell differentiation occurred on the 4th day, and the cells reached maturation on the 5th day. The SV40 tsA58 gene was active under nonpermissive temperature conditions but became completely inactivated upon thermoswitching to 39 °C. To determine the stability of the MPCs, the expression of the SV40 tsA58 gene was detected using RT-qPCR. There was no significant difference between undifferentiated and differentiated MPCs, which indicated that the SV40 tsA58 gene was normally expressed in MPCs (Fig. 4G). The first amplified monoclonal cell line was defined as the first passage (P1) of the MPCs. We compared WT1 and synaptopodin expression at P10, P15, and P20 in differentiated podocytes using Western blot analysis. The expression of WT1 and synaptopodin at P20 was significantly lower than at P10 (Fig. 4H–J). MPCs before P20 may be used for the study of podocyte functions and mechanisms.

To determine the quality of the MPCs, we observed the status of two mouse podocyte cell lines under an optical microscope over time (Fig. 4K). We found that the MPCs had already differentiated into foot processes on day 5, but the existing mouse podocyte cell line differentiated on day 7. The longer the MPCs survived in vitro, the more foot processes appeared, and the larger the cell volume became. The morphology of the MPCs also closely resembled that of mature podocytes found in vivo*.* MPCs maintained at 39 °C survived for 30 days, but the existing mouse podocyte cell line was essentially apoptotic by day 30 at 37 °C. This finding demonstrated that the established immortalized MPC line had a prolonged in vitro survival time and exhibited robust cell viability. In conclusion, MPCs demonstrated outstanding performance that was characterized by a brief differentiation period and prolonged in vitro survival.

### In vitro high glucose-induced podocyte injury model

**Figure 5:**
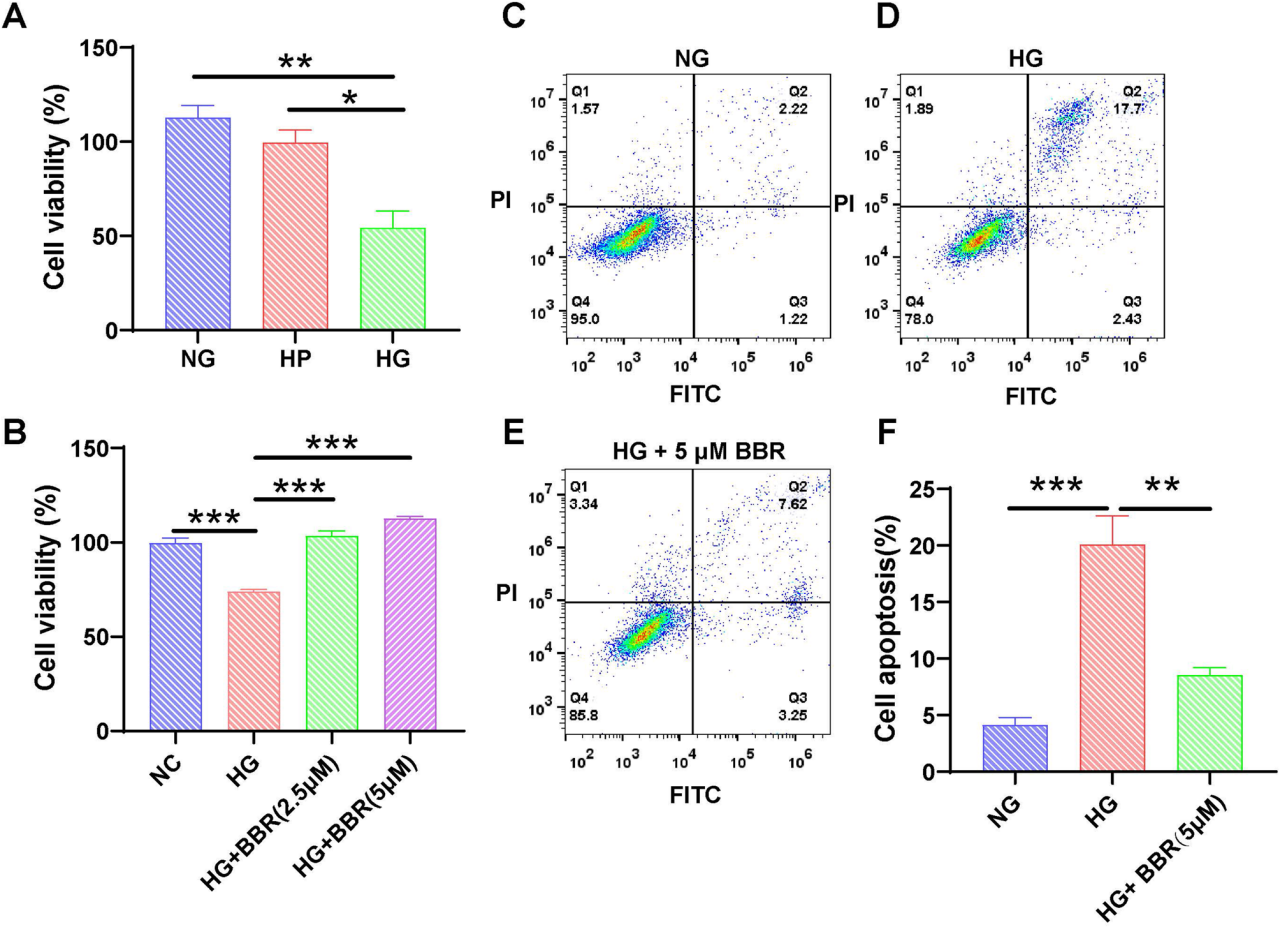
In vitro model of podocyte injury induced by high glucose. (**A**) Viability analysis using the CCK-8 assay showed a reduction in cell viability after treatment with HG. NG, normal glucose; HP, high permeability; HG, high glucose. (**B**) CCK-8 viability analysis showed alleviation of HG-induced MPCs after BBR treatment. Apoptosis of MPCs was analyzed using flow cytometry: (**C**) NG, (**D**) HG, and (**E**) HG + 5 mM BBR. (**F**) The percentage of apoptotic MPCs was determined as the average of three independent experiments. The data are presented as the means ± SEM (n = 3–6), *P < 0.05, **P < 0.01, ***P < 0.001.

To simulate the injurious state of podocytes in the DN, we induced podocyte injury in vitro using HG stimulation^6,52,53^. Comparative analysis with the normal glucose and high-permeability groups revealed a significant reduction in cell viability in the HG-induced MPCs (Fig. 5A), which indicated that HG led to MPC injury. The natural product berberine (BBR) effectively mitigates HG-induced apoptosis in podocytes, and it is valued in various countries^54^. BBR can reduce inflammation inhibit TLR4/NF-κB signaling pathway, and reduce HG-induced apoptosis in podocytes^55^. BBR reduces the phosphorylation of P70S6K and 4EBP1 by inhibiting mTOR/P70S6K/4EBP1 signaling pathway, increases podocyte autophagy, and decreases HG-induced apoptosis in podocytes^53^. To further validate the reliability of the MPC model, we initially examined the alleviating effect of BBR on podocyte injury. Compared to the normal control group, the viability of podocytes in the HG group significantly increased in a dose-dependent manner upon BBR treatment (Fig. 5B). We evaluated the impact of BBR on HG-induced podocyte apoptosis using flow cytometry. In the normal glucose group, the percentage of apoptotic MPCs was 2.22% (Fig. 5C), and the percentage increased to 17.7% in the HG group (Fig. 5D). Remarkably, BBR treatment in the HG group reduced the percentage of apoptotic MPCs to 7.62% (Fig. 5E). Each group was subjected to three independent replicates, and the apoptosis rates were statistically analyzed (Fig. 5F). BBR distinctly mitigated HG-induced MPC apoptosis, which is consistent with existing research^53,56^.

### In vitro ADR-induced podocyte injury model

**Figure 6:**
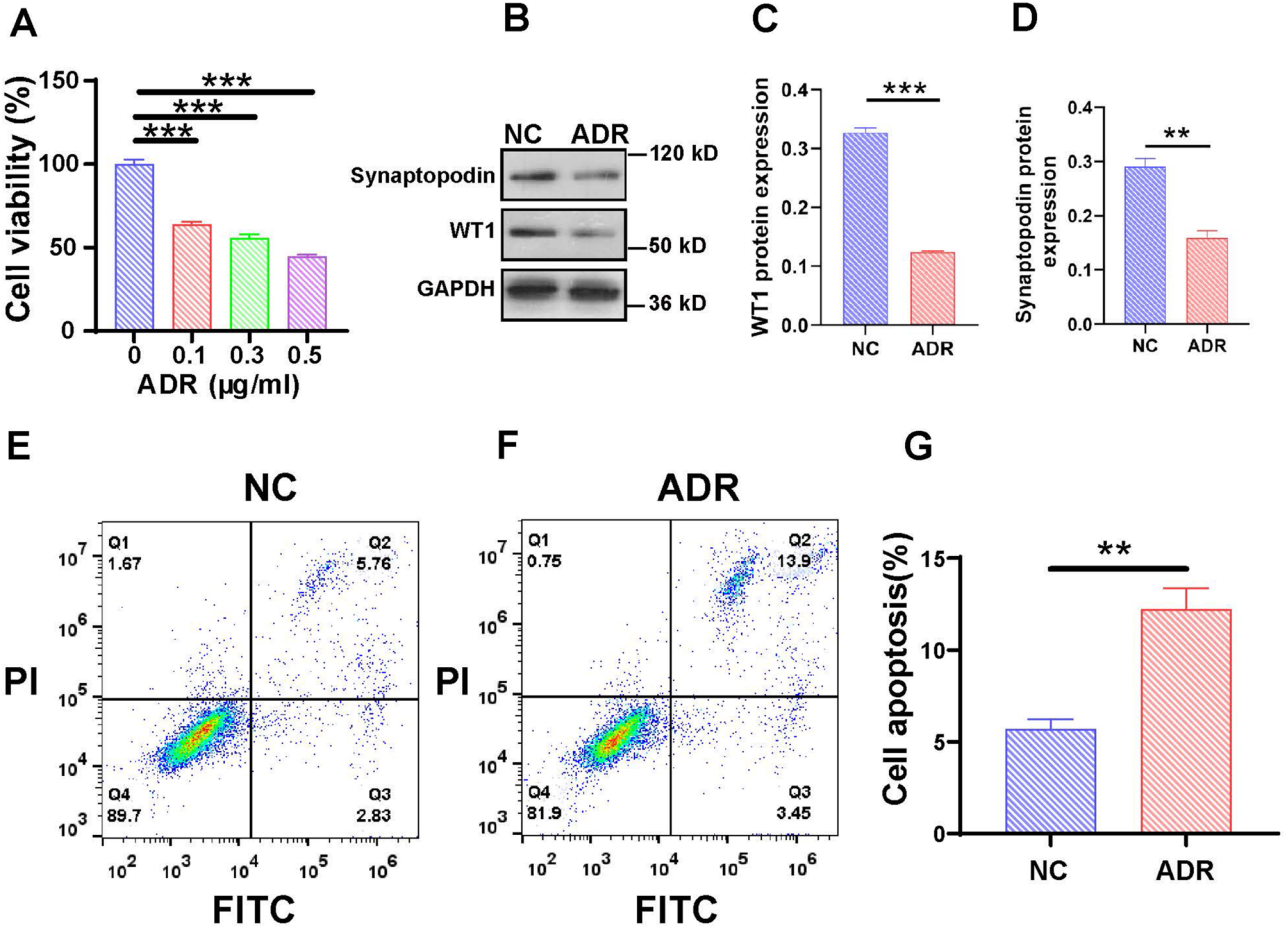
Induction of podocyte injury model using ADR in vitro. (**A**) CCK-8 viability analysis showed a reduction in cell viability after treatment with ADR. (**B**) Changes in WT1 and synaptopodin expression in the NC and ADR groups. (**C**,**D**) Intensity analyses of WT1 and synaptopodin in (**B**) using ImageJ. The apoptosis of MPCs was analyzed using flow cytometry (**E**) NC and (**F**) ADR. (**G**) The percentage of apoptotic MPCs was determined as the average across three independent experiments. The data are presented as the means ± SEM (n = 3–6), **P < 0.01, ***P < 0.001.

To simulate the state of podocyte loss and podocyte-specific protein downregulation, we induced podocyte injury using ADR. Compared to the normal control (NC) group, cell viability significantly decreased following ADR treatment, and cell viability progressively decreased with increasing concentrations of ADR (Fig. 6A). The expression of WT1 and synaptopodin was assessed using Western blotting. Compared to the normal control group, the number of ADR-induced MPCs was significantly lower (Fig. 6B–D). In certain glomerular diseases, a reduction in the number of podocytes is attributed to apoptosis^57^. In the normal control group, the percentage of apoptotic cells was 5.76% (Fig. 6E). Conversely, the percentage of apoptotic MPC cells increased to 13.9% in the ADR-treated group (Fig. 6F). Each group was subjected to three independent replicates, and the apoptosis rates were subjected to statistical analysis (Fig. 6G). Our findings incontrovertibly demonstrated that ADR significantly induced MPC apoptosis, which is consistent with previous research^58^.

### In vitro LPS-induced podocyte injury model

**Figure 7:**
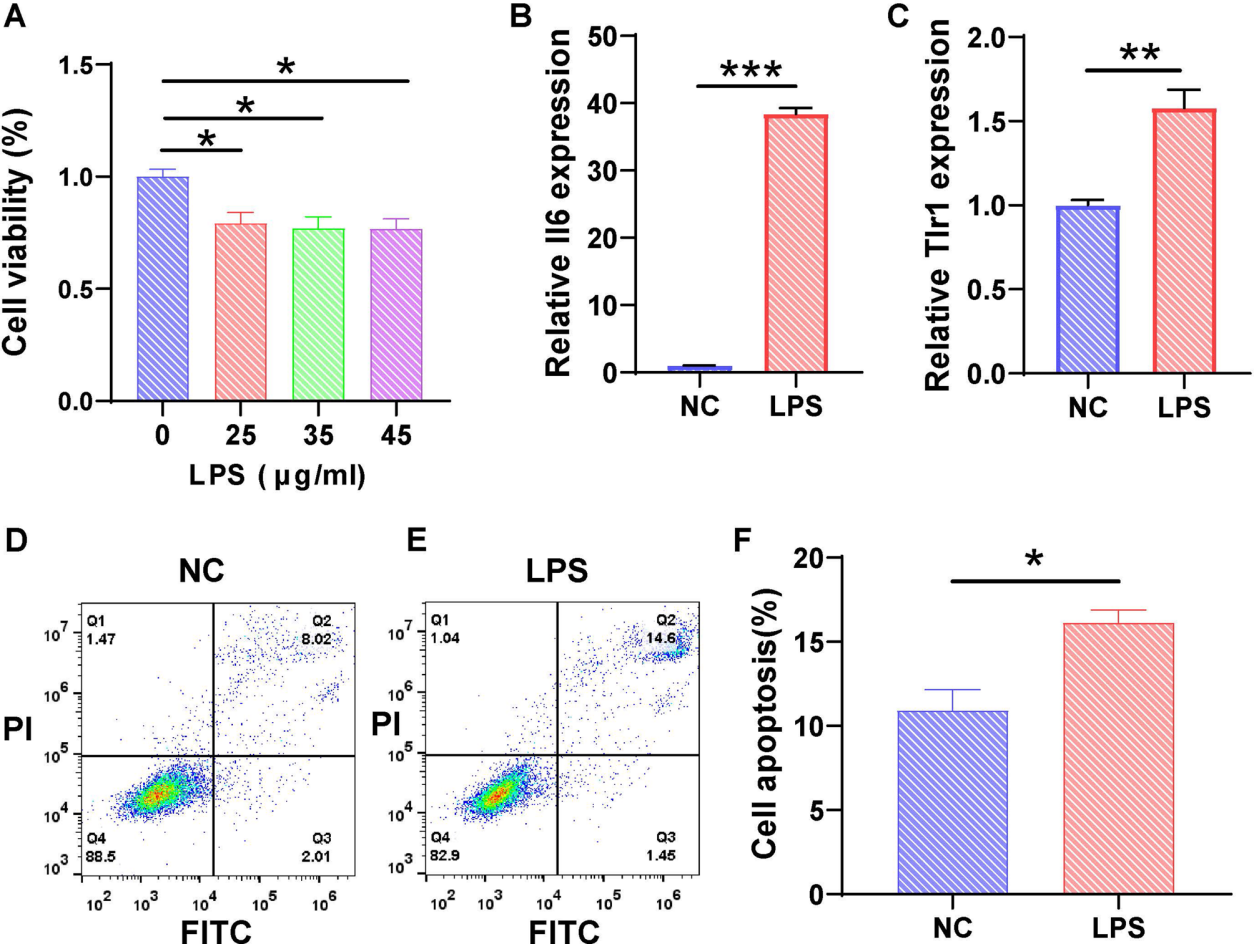
Induction of the podocyte injury model using LPS in vitro. (**A**) Viability analysis using the CCK-8 assay showed a reduction in MPC viability after treatment with LPS. (n = 6) (**B**,**C**) RT‒qPCR analysis of *Il6* and *Tlr1* expression in the NC and LPS groups. Apoptosis of MPCs was analyzed using flow cytometry following treatment with (**D**) NC or (**E**) LPS. (**F**) The percentage of apoptotic MPCs was determined as the average across three independent experiments. The data are presented as the means ± SEM (n = 3–6), *P < 0.05, **P < 0.01, ***P < 0.001.

To investigate podocyte immune injury within the glomerulus, we induced immune-related podocyte injury with LPS. We observed a significant reduction in the viability of LPS-treated MPCs compared to NC MPCs, which decreased further with increasing LPS concentrations (Fig. 7A). To assess the inflammatory response triggered by LPS, we performed RT-qPCR analysis to measure the mRNA expression of *Il6* and *Tlr6* in the MPCs (Fig. 7B,C). In contrast to the NC group, the expression of *Il6* and *Tlr6* mRNAs increased in LPS-induced MPCs, which indicated the stimulation of an immune response within the MPCs. To evaluate the impact of LPS on MPC apoptosis, flow cytometry was used to measure the percentage of apoptotic cells. In the NC group, the MPC apoptosis rate was 8.02% (Fig. 7D), but it increased to 14.6% in the LPS-treated group (Fig. 7E). Each group underwent three independent replicates, and statistical analysis of the percentages of apoptotic cells was performed (Fig. 7F). The results unequivocally demonstrated that LPS induced MPC apoptosis. Our findings are consistent with the conclusions drawn from the literature^58^.

## Discussion

Our study improved glomerular harvesting using suckling mouse kidneys. We generated conditionally immortalized MPCs via infection with the SV40 tsA58 gene. The appearance and functionality of the differentiated MPCs closely resembled typical glomerular podocytes under physiological conditions. Notably, we successfully established an MPC line with a shorter differentiation period and an extended in vitro survival time. Alternative approaches exist for obtaining podocytes, including derivation from urine^59^ and the differentiation of induced pluripotent stem cells (iPSCs) into podocytes^60^. However, podocytes obtained from urine may suffer damage and not accurately represent the physiological state of podocytes^59^. Podocytes induced from iPSCs also exhibit disparities compared to normal podocytes and face challenges in achieving widespread implementation^60^.

Podocytes were initially identified using epithelial or endothelial cell-specific markers^9,22,61–65^, but these markers lacked specificity for podocytes. In vitro cultivation of podocytes is hindered by rapid dedifferentiation, which results in the loss of structural and functional characteristics^30^ and further complicates their identification. Synaptopodin was the first protein recognized as a specific marker for podocytes, and it plays a vital role in maintaining the stability of the podocyte cytoskeleton^66,67^. Notably, synaptopodin is exclusively expressed during the mature stage of human kidney development, which highlights its significance as a marker for mature human podocytes^34^. Xinyi Yu et al. induced podocytes proliferation and differentiation at 37 °C via infection with SV40 TAg containing the FRT locus, albeit without the expression of Synaptopodin^68^. Another key biomarker, WT1, which is known for its regulation of kidney development, is exclusively expressed in mature podocytes within the kidney^69,70^. These two classical biomarkers are sufficient to characterize podocytes. We selected a monoclonal cell line that most resembled podocytes by excluding other types of glomerular cells using gene expression, and WT1 and synaptopodin were not the most abundant in cell line No. 5. At permissive temperatures, undifferentiated podocytes are similar to immature podocytes in vivo, and synaptopodin is expressed at low levels in immature podocytes. Therefore, the expression of synaptopodin in podocytes was not significantly different from MKCs.

We established immortalized MPCs in which *NPHS1* was minimally expressed. Consistent with previous investigations on other established podocyte lines, NPHS1 expression was not detected^71^. *NPHS2* and *THSD7A* are specifically expressed in SD^39,40,72^ and exhibit the same expression pattern as *NPHS1*. This expression may be because the formation of the SD requires a more stringent environment, such as in vivo. It is difficult to mimic the physiological environment in an in vitro culture, which resulting in the inability to form a stable structure for the SD and decreased expression of the SD proteins.

MPCs were stimulated by HG, ADR, or LPS to simulate injuries caused by different factors. Within the injured MPCs, alterations in proteins, inflammatory cytokines, and apoptosis were discerned. DN is a principal complication in diabetic patients, and podocyte injury occurs even in the early stages^73^. Consequently, HG-induced MPC injury is pivotal for early intervention and prophylaxis of DN. Induction of MPC injury using ADR led to the downregulation of WT1 and synaptopodin. The decreased expression of podocyte-specific proteins corresponded to the findings in patients with nephrotic syndrome^74^. Elevated expression of the inflammatory cytokines *Tlr1* and *Il6* indicates an inflammatory response in podocytes. These results demonstrate the successful simulation of podocyte injury in humans and highlight the utility of these models for exploring therapeutic targets in glomerular diseases, which further validate the reliability of the MPC model.

## Conclusion

In summary, we presented a universally applicable and streamlined method for establishing an MPC model that has significant advantages for in vitro investigations of podocytes and expansion of the existing repertoire of podocyte resources. This approach is a valuable and potent tool for advancing future research on podocytes.

### Supplementary Information

Supplementary Figures.

## Data Availability

The data that support the findings of this study are available upon reasonable request from the corresponding author.
