# Supplementary material for: A simple protocol to establish a conditionally immortalized mouse podocyte cell line
Source: Sci Rep. 2024 May 21;14:11591. doi: 10.1038/s41598-024-62547-5 (PMC11109129; doi:10.1038/s41598-024-62547-5)

## Supplementary data

**Supplementary Figure 1**


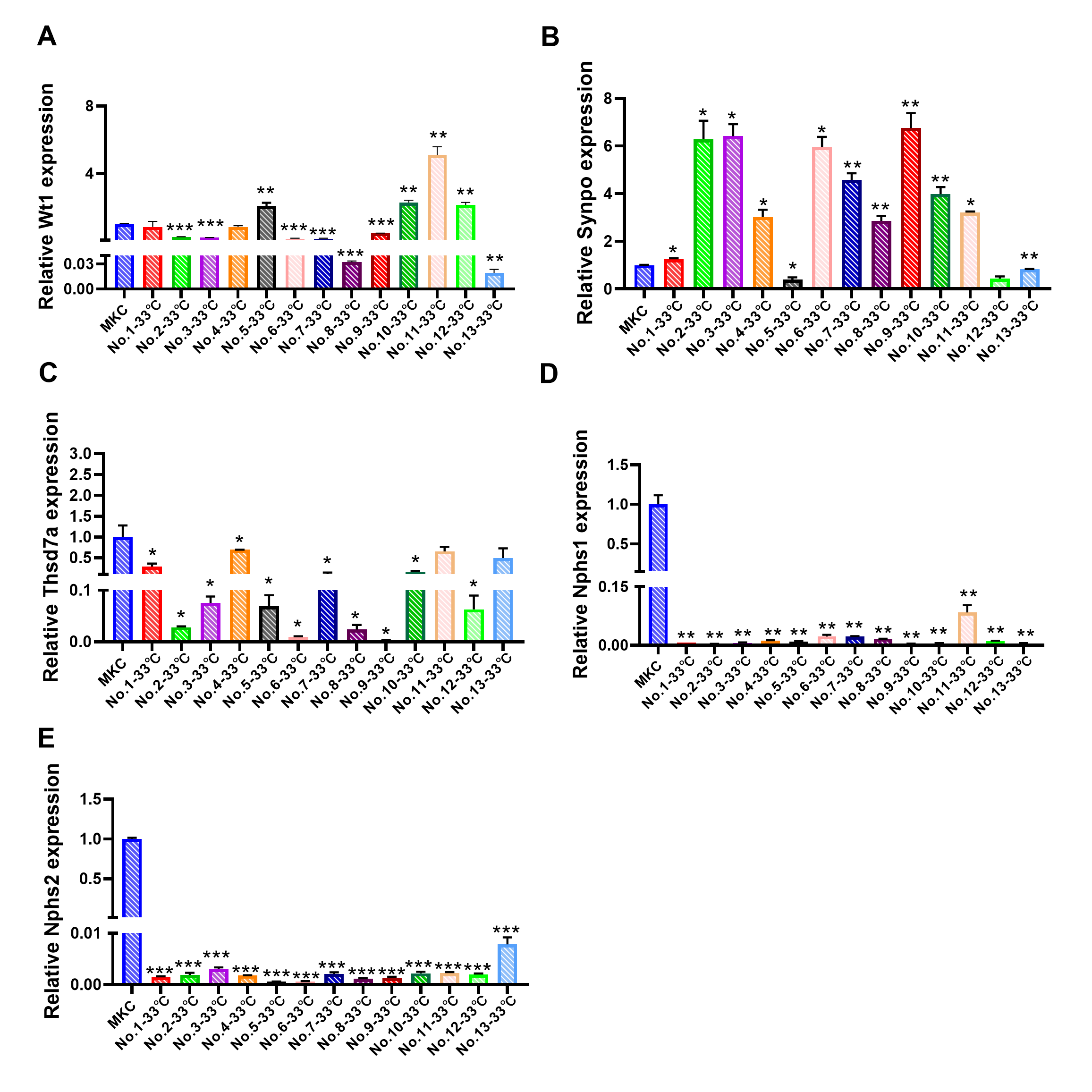


**Supplementary Figure 1| Expression of podocyte-specific markers detected by RT-qPCR in 13 monoclonal cell lines.** MKC was used as a control. (A) Expression of *Wt1* in 13 monoclonal cell lines. (B) The expression of *Synpo* in 13 monoclonal cell lines. (C) The expression of *Thsd7a* in 13 monoclonal cell lines. (D) Expression of *Nphs1* in monoclonal cell lines. (E) Expression of *Nphs2* in monoclonal cell lines. The data are presented as the means ± SEM (n=3), ** *P* <0.05, ** *P* <0.01, ****P* < 0.001.

**Supplementary Figure 2**


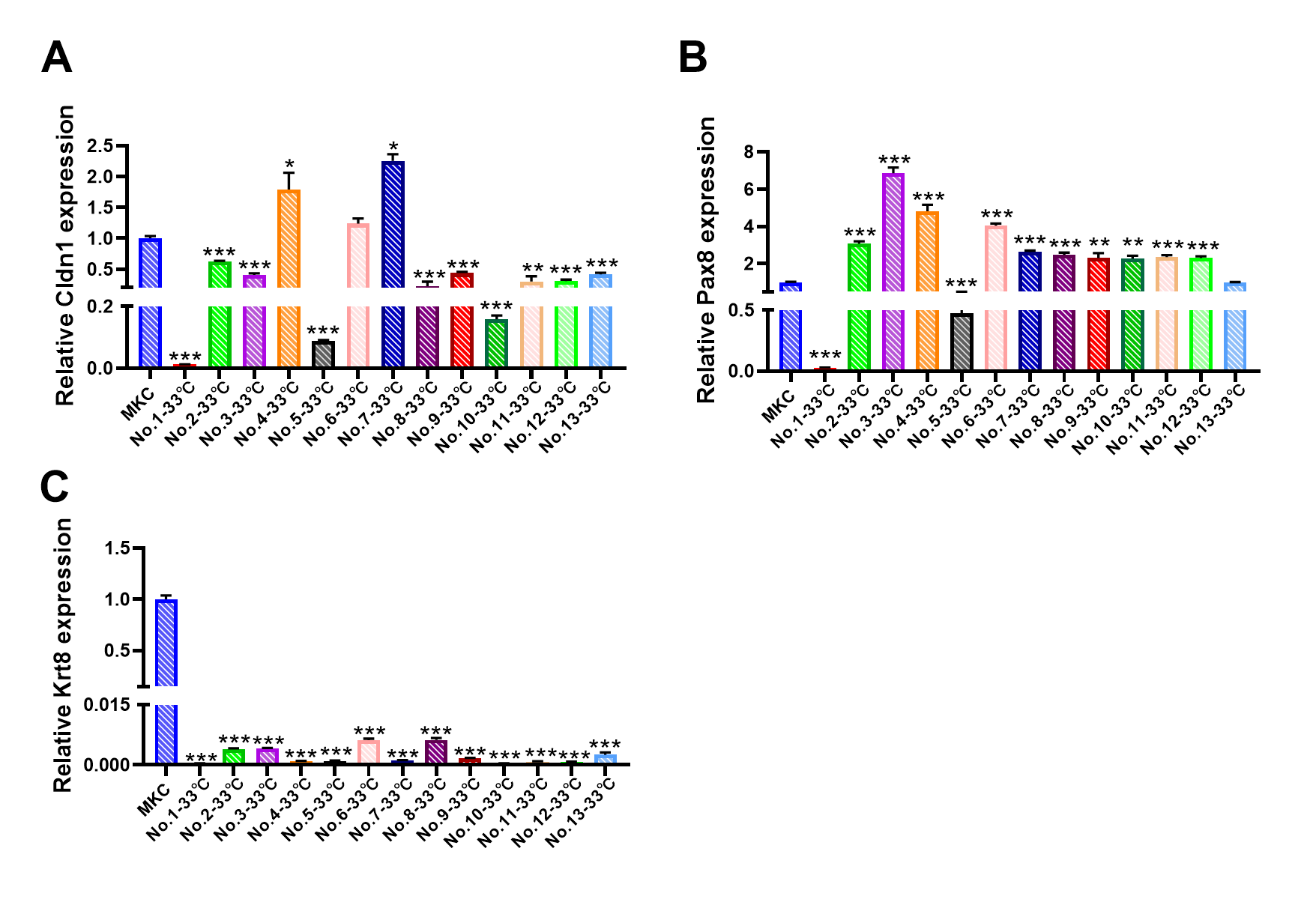


**Supplementary Figure 2| Expression of PEC-specific markers detected by RT-qPCR in 13 monoclonal cell lines.** MKC was used as a control. (A) Expression of *Cldn1* in 13 monoclonal cell lines. (B) The expression of *Pax8* in monoclonal cell lines. (C) The expression of *Krt8* in monoclonal cell lines. The data are presented as the means ± SEM (n=3), * *P* <0.05, ** *P* <0.01, *** *P* < 0.001.

**Supplementary Figure 3**


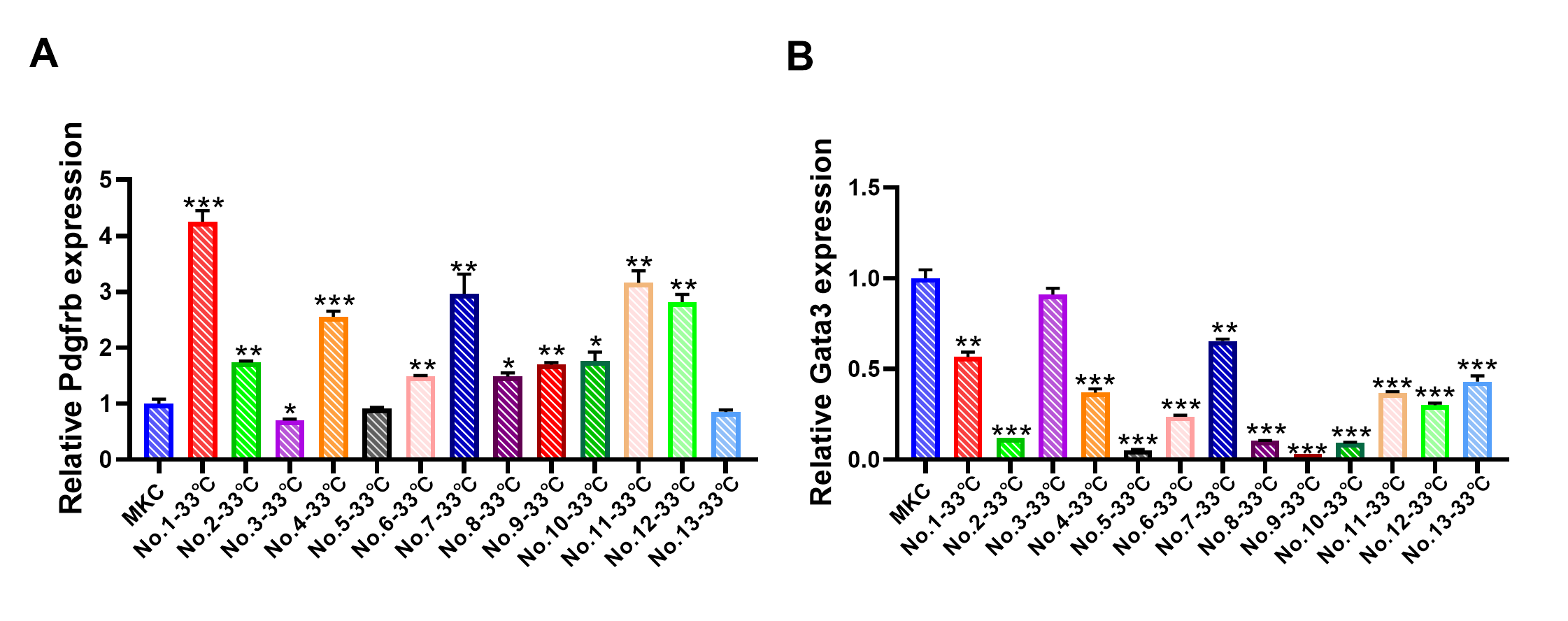


**Supplementary Figure 3| Expression of mesangial cell-specific markers detected by RT-qPCR in 13 monoclonal cell lines**. MKC was used as a control. (A) The expression of *Pdgfrb* in 13 monoclonal cell lines. (B) The expression of *Gata3* in 13 monoclonal cell lines. The data are presented as the means ± SEM (n=3), * *P* <0.05, ** *P* <0.01, *** *P* < 0.001.

**Supplementary Figure 4**


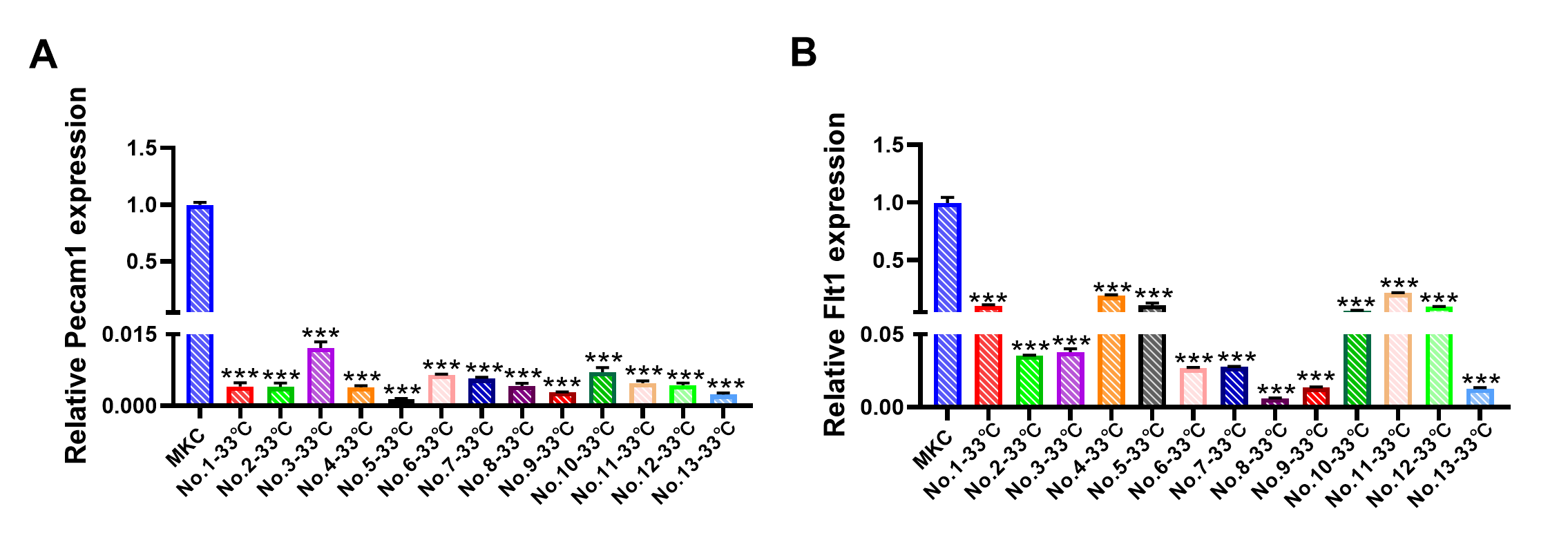


**Supplementary Figure 4| Expression of** **endothelial cell-specific markers detected by RT-qPCR in 13 monoclonal cell lines.** MKC was used as a control. (A) Expression of *Pecam1* in monoclonal cell lines. (B) Expression of *Flt1* in 13 monoclonal cell lines. The data are presented as the means ± SEM (n=3), *** *P* < 0.001.

**Supplementary Figure 5**


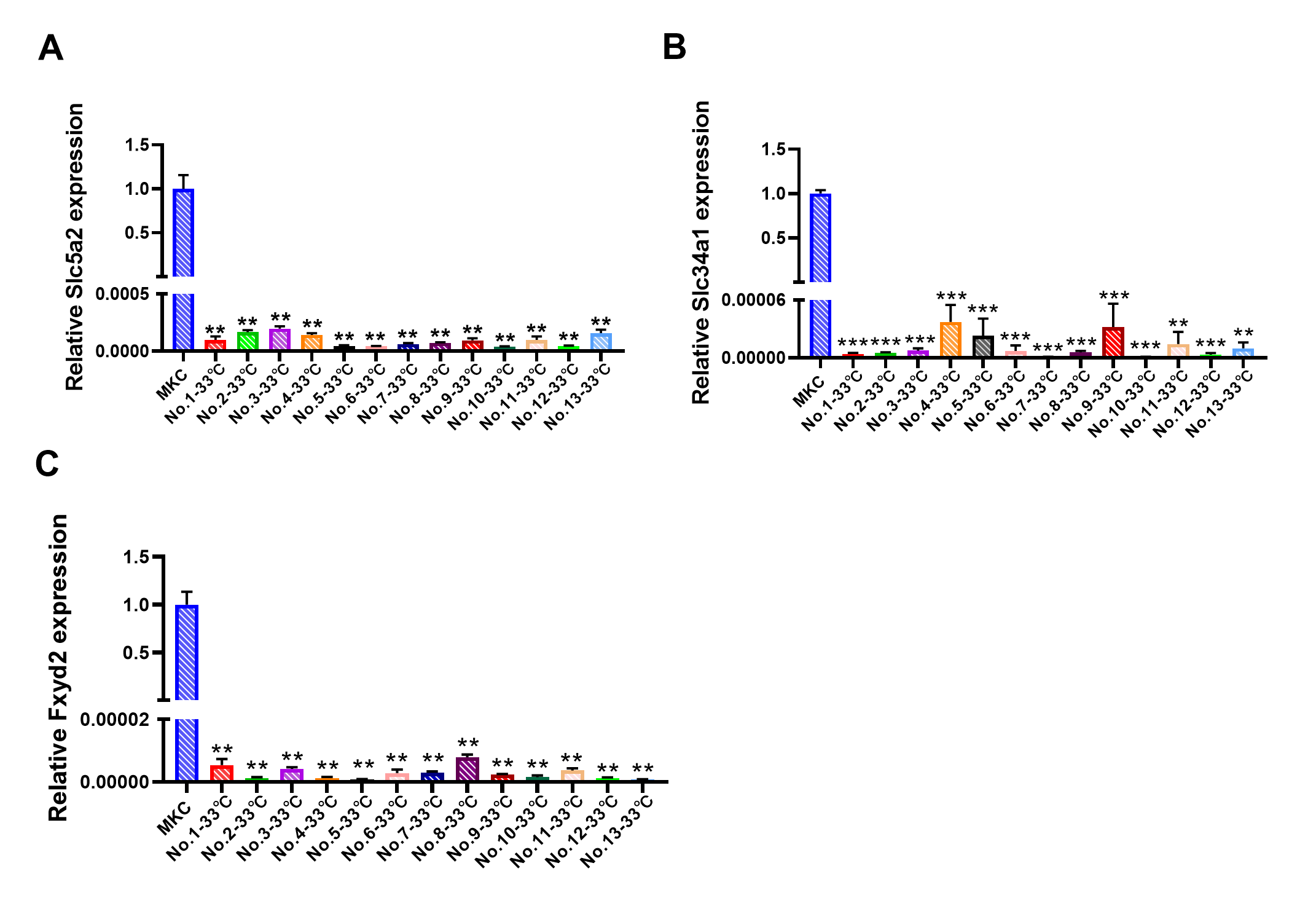


**Supplementary Figure 5| Expression of renal tubular epithelial cell-specific markers detected by RT-qPCR in 13 monoclonal cell lines.** MKC was used as a control. (A) The expression of *Slc5a2* in 13 monoclonal cell lines. (B) The expression of *Slc34a1* in 13 monoclonal cell lines. (C) *Fxyd2* expression in 13 monoclonal cell lines. The data are presented as the means ± SEM (n=3), ** *P* <0.01, *** *P* < 0.001.

**Supplementary Figure 6**


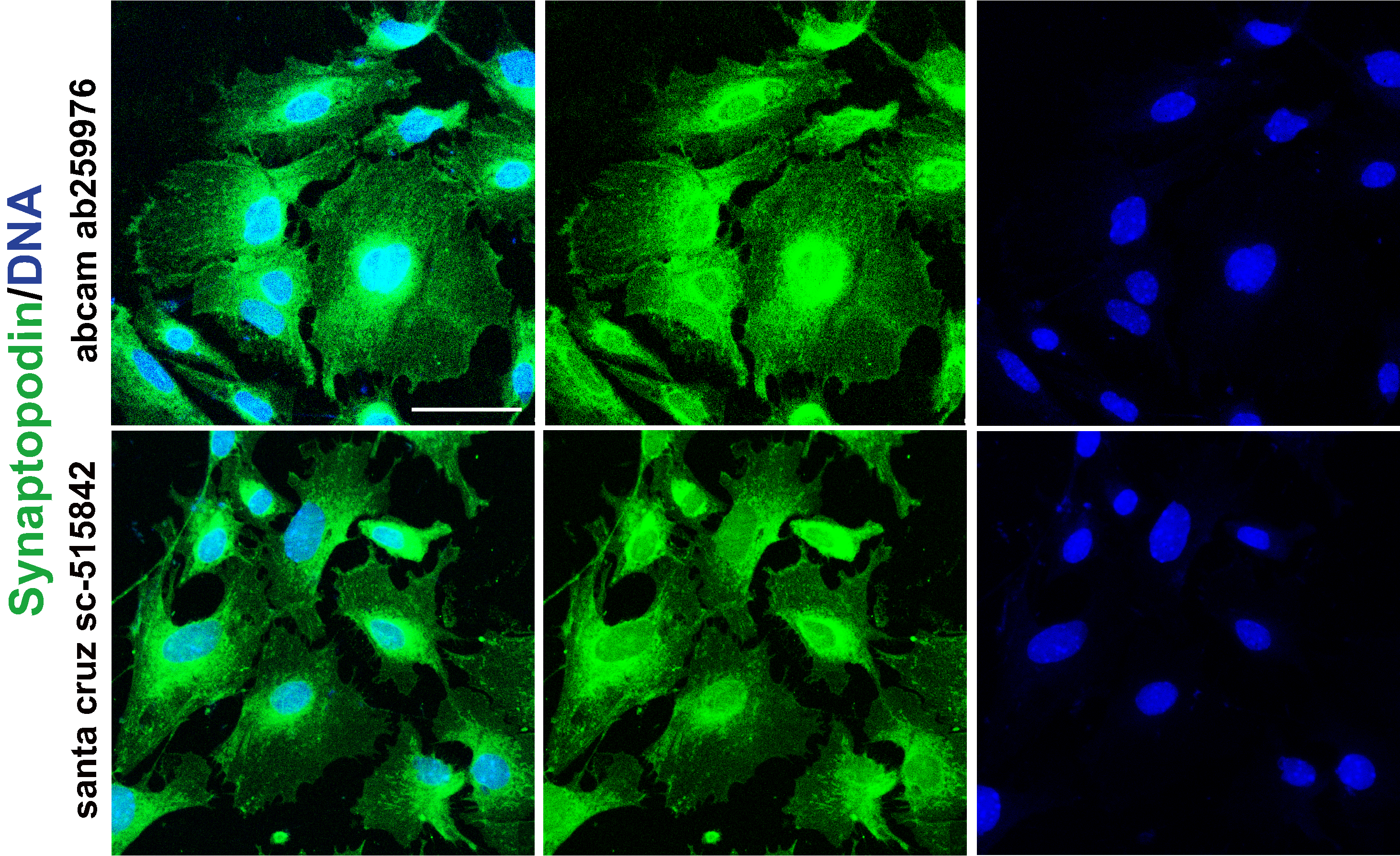


**Supplementary Figure 6|** **Indirect immunofluorescent staining showed two other primary anti-synaptopodin antibodies staining in glomerular cells.** The top was the staining of manufacturer Abcam, and the bottom was the staining of manufacturer Santa Cruz.

**Supplementary Figure 7**


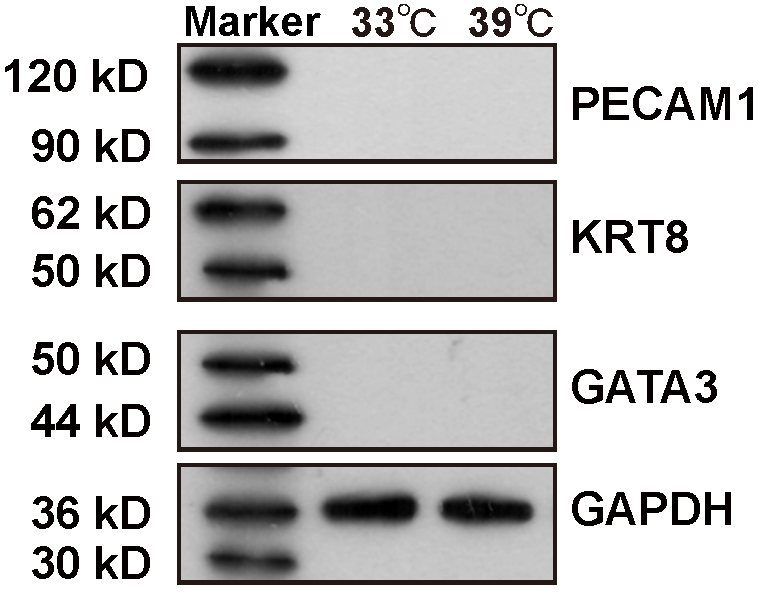


**Supplementary Figure** **7|** **Expression of PEC-specific protein,** **mesangial cell-specific protein, and endothelial cell-specific protein detected in the No. 5 cell line**. PECAM1 is an endothelial cell-specific protein. KRT8 is a PEC-specific protein. GATA3 is a mesangial cell-specific protein.

**Western blot original**

**Figure 4B Synaptopodin**


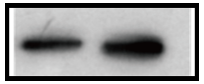

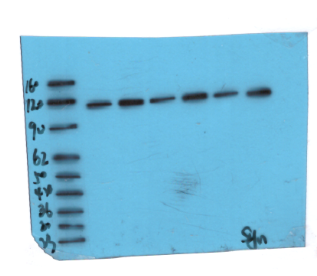


**Figure 4B WT1**


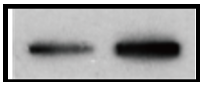

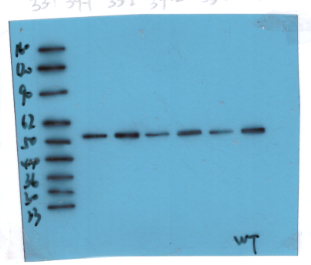


**Figure 4B GAPDH**


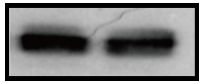

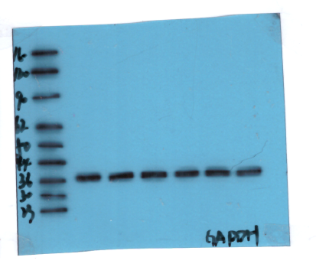


**Figure 4F SV40 Tag**







**Figure 4F GAPDH**







**Figure 4H Synaptopdin**


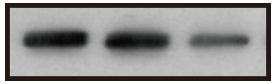

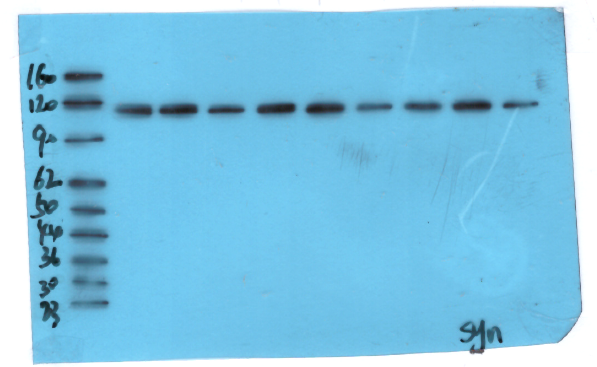


**Figure 4H WT1**


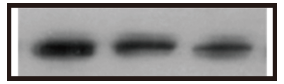

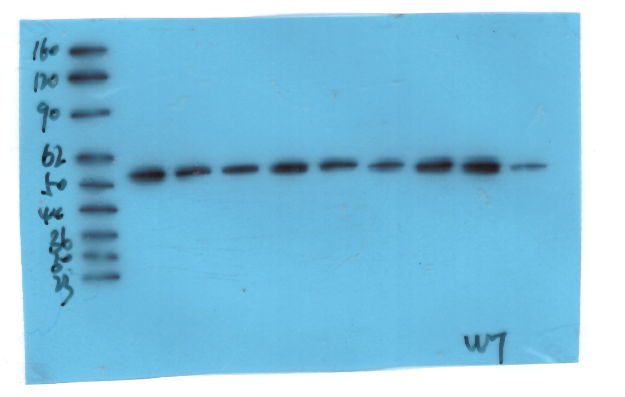


**Figure 4H GAPDH**


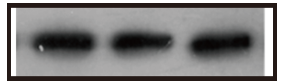

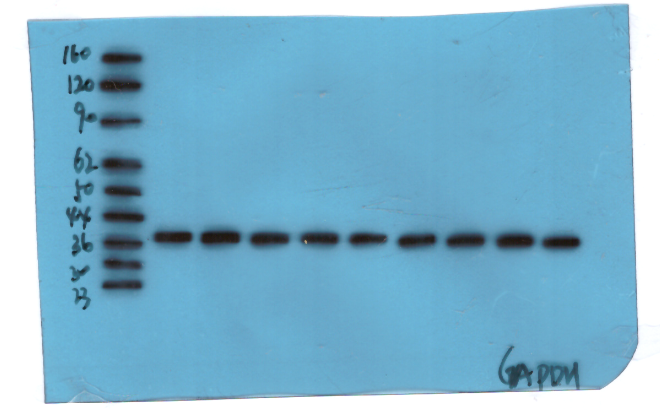


**Figure 6B Synaptopodin**


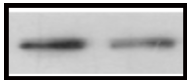

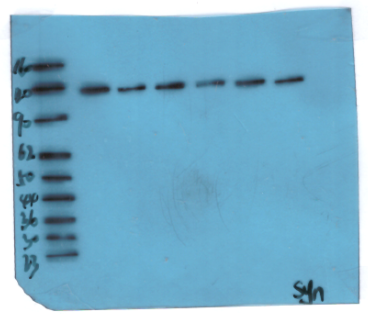


**Figure 6B WT1**


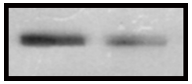

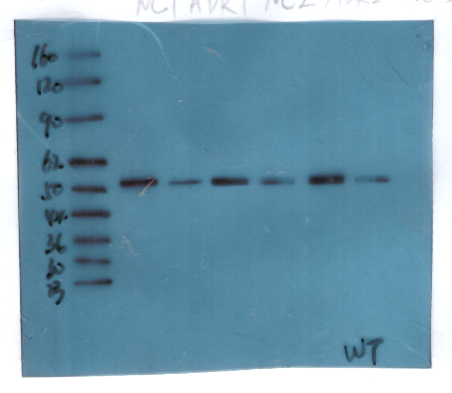


**Figure 6B GAPDH**


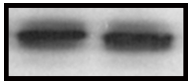

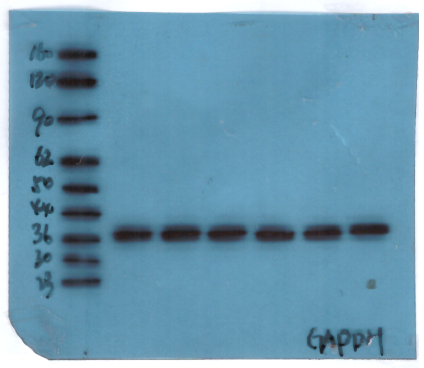


**Supplementary Figure 7 PECAM1**


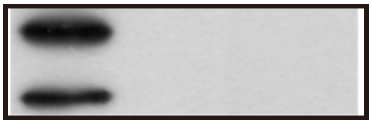

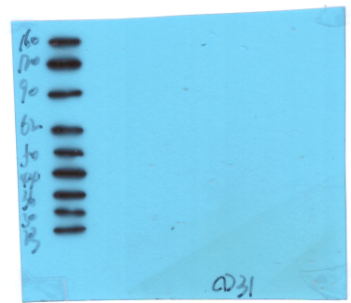


**Supplementary Figure 7 GATA3**


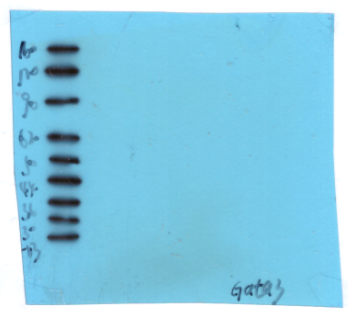


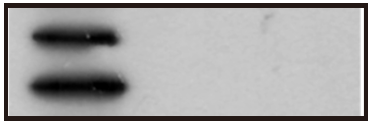


**Supplementary Figure 7 KRT8**


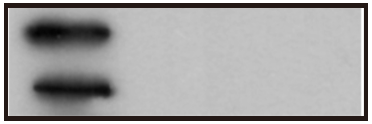

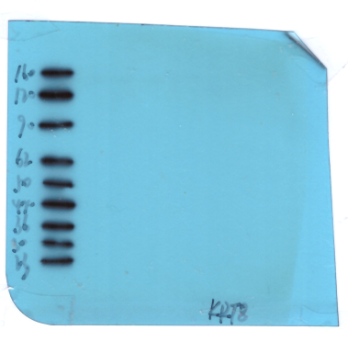


**Supplementary Figure 7 GAPDH**

**
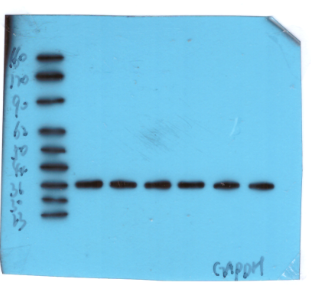
**


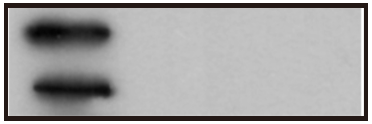

Supplement: Supplementary file 1 — Supplementary Figures. [file 41598_2024_62547_MOESM1_ESM.docx]
